# International comparison of availability for orphan drugs: focused on approved orphan drugs in South Korea

**DOI:** 10.1080/20523211.2024.2354299

**Published:** 2024-06-05

**Authors:** Eun Young Shin, Young Jun Hong, Kyung Min Lim, Tae Hyang Kim, Jong Hyuk Lee

**Affiliations:** College of Pharmacy, Chung-Ang University, Seoul, Republic of Korea

**Keywords:** Orphan drugs, rare disease, availability, approval time, drug lag, designation gap

## Abstract

**Introduction:**

In this study, we aimed to comparatively analyse the indicators of availability to orphan drugs in South Korea, the United States of America, Europe Union, and Japan.

**Methods:**

For 169 drugs designated as orphan drugs in South Korea between 2012 and 2021, information on the drugs designated as orphan drugs from each jurisdiction was extracted by country. Then, the availability indicators (approval time, drug lag time, and designation gap) were analysed for the drugs approved in each jurisdiction.

**Results:**

The approval rate of drugs designated as orphan drugs were 11.22% and 6.31% in the USA and EU, respectively, which was lower than that of orphan drugs in South Korea and Japan. The highest number of approved drugs was in the USA (87 drugs), EU 27 drugs, Japan 22 drugs and Korea 21 drugs. Furthermore, the approval time significantly differed between South Korea and the other countries. South Korea had a significantly different drug lag time and designation gap compared with the USA and EU.

**Conclusion:**

Our findings show that to fundamentally improve the access to treatments for rare disease, a policy of regulatory science that can comprehensively support the early stages of research and development and commercialisation is needed.

## Introduction

Owing to their substantially low incidence, there is an insufficient number of patients available for clinical trials required to develop treatments for rare diseases. This presents numerous obstacles in the research and development (R&D) process, resulting in limitations when validating the safety and efficacy of conventional clinical trial designs (Cavazzoni, 2022b). Without assurance of adequate compensation during the R&D and commercialisation phases, pharmaceutical companies may hesitate to actively participate in the development of new drugs for rare diseases because of the uncertainty regarding recovering their investments (Roberts & Wadhwa, 2023). Given that issues of patient access to orphan drugs are related to various social aspects, including resource distribution and ethical concerns, legislation was introduced in each country to improve access to these drugs (Rodriguez-Monguio et al., 2017). Particularly, availability is one of the components of access to medicine, which is a factor related to drug research and development and approval of drugs to the market, and in this regard, the United States of America (USA) implemented the Orphan Drug Act in 1983 to provide various benefits to the R&D of orphan drugs (Kim & Kim, 2018; WHO, 2006). Similarly, major countries, such as Japan (Pharmaceutical Affairs Law) and those in Europe Union (EU) (Regulation EC/141/2000), have established laws to promote R&D of orphan drugs (Harada et al., 2020; Wellman-Labadie & Zhou, 2010). In South Korea, the ‘Regulations on the Designation of Orphan Drugs’ was enacted in 1998 to stipulate the designation standards and procedures for orphan drugs. Additionally, with the enforcement of the Rare Disease Control Act in 2016, a legal framework was established to offer administrative and financial support for the production and sale of orphan drugs. While each jurisdiction has different designation criteria and benefits for rare diseases, they generally include economic incentives, expedited reviews, and market exclusivity (Table 1) (Center, K. L. I., 2018, 2020; CODE, U. S., 2023; EMA, 2023a, 2023c; FDA, 2022a, 2023a; GAO, U. S., 2020; Huang et al., 2022; MHLW, 2023; Nagai, 2019; Srivastava & Winslow, 2019). In other words, the designation of orphan drugs entails policy support throughout the entire drug development cycle, from R&D to new drug application (NDA) to the post-marketing stage. Therefore, it is crucial to implement related systems harmoniously at each stage to ensure that access to orphan drugs aligns with the healthcare environment of each jurisdiction. Recently, owing to policy support from each jurisdiction as well as the development of medical technology, R&D investments in orphan drugs have increased worldwide, and the number of approved orphan drugs is increasing annually. In 2021, 26 of the 50 new drugs approved in the USA were orphan drugs, all designated as such from the development stage (Cavazzoni, 2022a; Hadjivasiliou, 2022).

**Table 1.** Criteria and incentives for orphan drug in South Korea and three major jurisdictions.

Terms		South Korea	USA	EU	Japan
Legal basis		The 1998 Regulation on Orphan Drug Designation Notification	The 1983 Orphan Drug Act and amendments	Regulation (EC) No 141/2000	Pharmaceutical Affairs Law
Prevalence of rare disease		< 20,000 patients	< 200,000 patients	< 5 in 10,000 patients	< 50,000 patients
Orphan drug designation authority		MFDS	OOPD (FDA)	COMP (EMA)	MLHW – PMDA – NIBIO
Incentives for orphan drug development	Research grants	✓	✓	✓	✓
	Tax incentives	✓	✓ (Tax credits for qualified clinical trials)	✓ (Incentives in Meer States)	✓ (12% of study expenses for orphan drug incurred during the NIBIO subsidy payment period can be reported as a tax credit)
	Fee waivers and reductions	✓ (Reduction of fee such as approval, priority review, and permission application)	✓ (There is no fee for human drug applications unless they include an indication other than a rare disease or condition)	✓ (Reduced fees for protocol assistance, marketing-authorisation applications, inspections before authorisation, applications for changes to marketing authorisations made after approval, and reduced annual fees)	✓ (PMDA provides a priority consultation system for designated orphan drug. Lower user fee categories for PMDA’s consultation are applicable to designated orphan drugs)
	Exclusivity	✓ 4–10 years market exclusivity	✓ Potential for 7 years of market exclusivity after approval	✓ 10 years market exclusivity	✓ The re-examination period for the drugs will be extended up to 10 years for drugs
	Expedited review process	Priority Review Partial exemption from approval submission data	Priority review Breakthrough therapy designation Accelerated approval Fast track designation	Accelerated assessment Conditional marketing authorisation Authorisation under exceptional circumstances	Priority review

Note: EC: European Commission; MFDS: Ministry of Food and Drug Safety; OOPD: Office of Orphan Products Development; COMP: Committee for Orphan Medicinal Products; MLHW: Ministry of Health, Labour, and Welfare of Japan; PMDA: Pharmaceuticals and Medical Devices Agency; NIBIO: National Institute of Biomedical Innovation

In a country that is highly dependent on the import of orphan drugs, such as South Korea, the time it takes for orphan drugs that have been developed and marketed overseas to be approved, that is, the drug lag time, has a great impact on availability of the drug, and furthermore patient access to medicine. Since availability to orphan drugs greatly worsens with the prolongation of the drug lag time, efforts to shorten the time through national policy support are required. Thus, comparing the various quantitative indicators related to the availability to orphan drugs with those of other countries may help determine how well related systems work systematically and evaluate their performance.

Previous studies on access to Korean orphan drugs are mostly related to the Pricing & Reimbursement (P&R) system. Although studies related to drug lag time in new drugs have been published, a study including international comparisons focused on the availability indicators of orphan drugs has not been conducted (Choi et al., 2023; Lee et al., 2020).

## Methods

### Data collection

Datasets for the designation and approval of orphan drugs in South Korea, the USA, EU, and Japan from 2012 to 2021 are established annually. A list of drugs designated and approved as orphan drugs in each jurisdiction was extracted from the official government agency sites (EMA, 2023d; FDA, 2023b; MFDS, 2023a, 2023b, 2023c; NIBIOHN, 2022; PMDA, 2023a, 2023b). A drug was classified as approved for products that matched the indications at the time of designation as an orphan drug and at the time of approval. Thus, a drug was not included in the list of approved drugs if the indication differed when approved from that designated as an orphan drug.

### Data analysis

Based on the 169 drugs designated as orphan drugs in South Korea between 2012 and 2021, designated orphan drugs from each jurisdiction were extracted (Korea: 169, USA: 130, EU: 102, Japan: 90). The following availability indicators were analysed for the drugs approved in each jurisdiction (South Korea: 116, USA: 120, EU: 87, Japan: 80) and in all four countries (45 drugs).

- Demanding time for marketing approval from designation (approval time): The period from the date of designation as an orphan drug to the date of approval.
- Drug lag time (drug lag): the period from the approval date of the first approved country to the date of approval in each country.
- Designation gap: Period from the designation date of the first designated country to the date of approval in each country.

In the drug lag time and designation gap analyses, the products that were first approved (Korea: 21, USA: 87, EU: 27, Japan: 22) or designated (Korea: 25, USA: 98, EU: 29, Japan: 17) in each trial were analysed by setting the value of the drug lag and designation gap to 0.

Descriptive statistics and the Mann–Whitney U test were used for statistical analysis (the significance level was set at *P* < 0.05). Statistical analyses were performed using SPSS Statistics Version 28 (IBM Corp).

## Result

### Orphan drug designation and approval status in South Korea and other countries

Between 2012 and 2021, 169, 3521, 1300, and 315 products were designated as orphan drugs in South Korea, the USA, EU, and Japan, respectively. As of December 31, 2021, 116, 395, 82, and 214 drugs were approved in Korea, USA, EU, and Japan, respectively, with the greatest number of drugs being approved in the USA. However, the approval rates (number of approved products/designated items) in order of highest to lowest rates was 68.64% (116/169), 67.94% (214/315), 11.22% (395/3,521), and 6.31% (82/1,300) in Korea, Japan, the USA, and EU, respectively (Table 2).

**Table 2.** Designation and approval status of orphan drugs in South Korea and three major jurisdictions countries (2012–2021).

Jurisdiction	Year/Classification	2012	2013	2014	2015	2016	2017	2018	2019	2020	2021	Total
South Korea	Designated Products^a^	10	13	16	27	18	3	16	21	19	26	169
	Approved Products^b^	10	11	15	21	15	3	14	14	13	0	116
	Approval Rate (%)	100.00	84.62	93.75	77.78	83.33	100.00	87.50	66.67	68.42	0.00	68.64
USA	Designated Products	207	278	302	367	353	492	343	340	476	363	3521
	Approved Products	56	73	48	53	60	54	23	15	12	1	395
	Approval Rate (%)	27.05	26.26	15.89	14.44	17.00	10.98	6.71	4.41	2.52	0.28	11.22
EU	Designated Products	89	87	152	134	167	129	144	98	137	163	1300
	Approved Products	17	11	15	10	7	9	10	2	1	0	82
	Approval Rate (%)	19.10	12.64	9.87	7.46	4.19	6.98	6.94	2.04	0.73	0.00	6.31
Japan	Designated Products	31	32	30	32	22	16	34	36	53	29	315
	Approved Products	26	26	25	27	20	14	27	26	22	1	214
	Approval Rate (%)	83.87	81.25	83.33	84.38	90.91	87.50	79.41	72.22	41.51	3.45	67.94

^a^ Designated products are the number of products designated as orphan drugs each year from 2012 to 2021.

^b^ Approved products are the number of approvals for products designated as orphan drugs when checked on December 31, 2021 (reference date). The current approval rate is low because it takes time to obtain approval after designation as an orphan drug.

### Analysis of approval time, lag time, and designation gap of orphan drugs approved in each jurisdiction

The designation and approval status of 169 drugs designated as orphan drugs in South Korea were analysed for each jurisdiction. Of the 169 drugs, 130, 102, and 90 were designated as orphan drugs in the USA, EU, and Japan, respectively, and among them, 120 (92.31%, 120/130), 87 (85.29%, 87/102), 80 (88.89%, 80/90), and 116 (68.64%, 116/169) were approved in the USA, EU, Japan, and South Korea, respectively. In the USA, 87 drugs were approved, which was the highest among the countries, while 27, 22, and 21 were approved in EU, Japan, and Korea, respectively.

**Figure 1.**
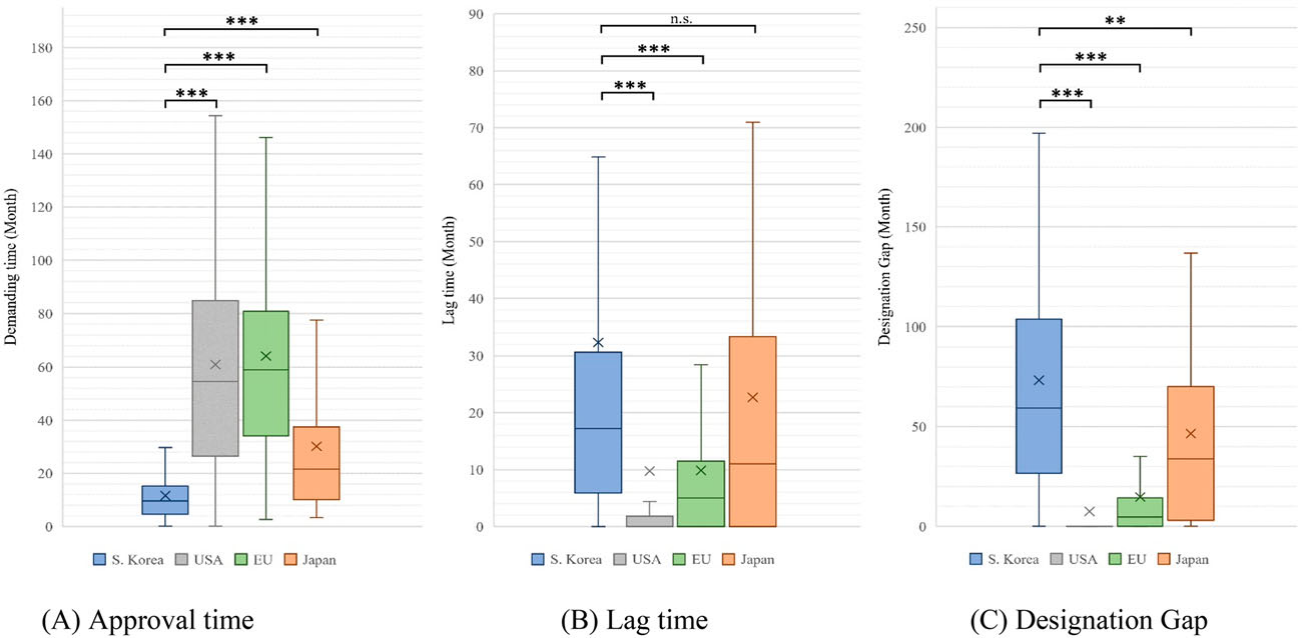
Approval time, lag time, and designation gap of orphan drugs approved in each jurisdiction. Note: Based on 169 products designated as orphan drugs in South Korea from 2012 to 2021. We compared the demand time, lag time, and designation gap by country for the products approved in each country. (A) Approval time: South Korea vs. USA, EU, and Japan (*P* < 0.001). (B) Lag time: South Korea vs. USA and EU (*P* < 0.001), and South Korea vs. Japan (*P* < 0.299). (C) Designation gap: South Korea vs. USA, EU (*P* < 0.001), and South Korea vs. Japan (*P* = 0.003). (We marked n.s.: not significant, *P < 0.05, **P < 0.01, and ***P < 0.001).

Subsequently, the approval time, lag time, and designation gap were analysed for products approved for each jurisdiction. In the analysis of the approval time, the results were as follows: South Korea (mean: 11.67 months, median: 9.70 months), USA (mean: 60.97 months, median: 54.55 months), EU (mean: 64.14 months, median: 59.00 months) and Japan (mean: 30.16 months, median: 21.65 months). The approval time differed significantly between South Korea and the other countries. (USA, EU, Japan: *P* < 0.001) (Table 3 and Figure 1).

**Table 3.** Approval time, lag time, and designation gap of orphan drugs approved in each jurisdiction.

Jurisdiction	Designated products (N)	Approved products (N)	Approval rate (%)	First designated products (N)	First approved products (N)	Approval time			Lag time			Designation gap		
						Mean	Median	*P* value	Mean	Median	*P* value	Mean	Median	*P* value
South Korea	169	116	68.64	15	21	11.67	9.70	–	32.36	17.23	–	73.34	59.34	–
USA	130	120	92.31	92	87	60.97	54.55	<0.001	9.73	0.00	<0.001	7.55	0.00	<0.001
EU	102	87	85.29	25	27	64.14	59.00	<0.001	9.86	5.00	<0.001	14.74	4.64	<0.001
Japan	90	80	88.89	17	22	30.16	21.65	<0.001	22.69	11.04	0.299	46.54	34.00	0.003

Note: Based on 169 products designated as orphan drugs in South Korea from 2012 to 2021. Approval time: Period (months) from designation to approval of drugs by country for products approved in each country. Lag time: Period (months) from the first country of approval to the approval of drugs by each country for products approved in each country. Designation gap: Period (months) from the first country of designation to the designation of drugs by country for products approved in each country. First approved and designated products: If the product was first approved or designated in the country, the drug lag and designation gap were set to zero. N: Number of designated (approved) products in each country. *P* Value: Compared with South Korea.

In the analysis of lag time, the results were as follows: South Korea (mean: 32.36 months, median: 17.23 months), USA (mean: 9.73 months, median: 0.00 month), EU (mean: 9.86 months, median: 5.03 months), and Japan (mean: 22.69 months, median: 11.04 months). Although there was no statistically significant difference between South Korea and Japan (*P* = 0.299), South Korea had a significantly different lag time compared with the USA and EU (USA, EU: *P* < 0.001) (Table 3 and Figure 1).

In the analysis of designation gap, the results were as follows: South Korea (mean: 73.34 months, median: 59.34 months), USA (mean: 7.55 months, median: 0.00 month), EU (mean: 14.74 months, median: 4.64 months), and Japan (mean: 46.54 months, median: 34.00 months). The designation gap in South Korea differed significantly from that of other countries (USA, EU: *P* < 0.001; Japan: *P* = 0.003) (Table 3 and Figure 1). The number of drugs designated by jurisdiction was 92 in the USA, followed by 25, 17, and 15 in EU, Japan, and South Korea, respectively (Table 3).

**Figure 2.**
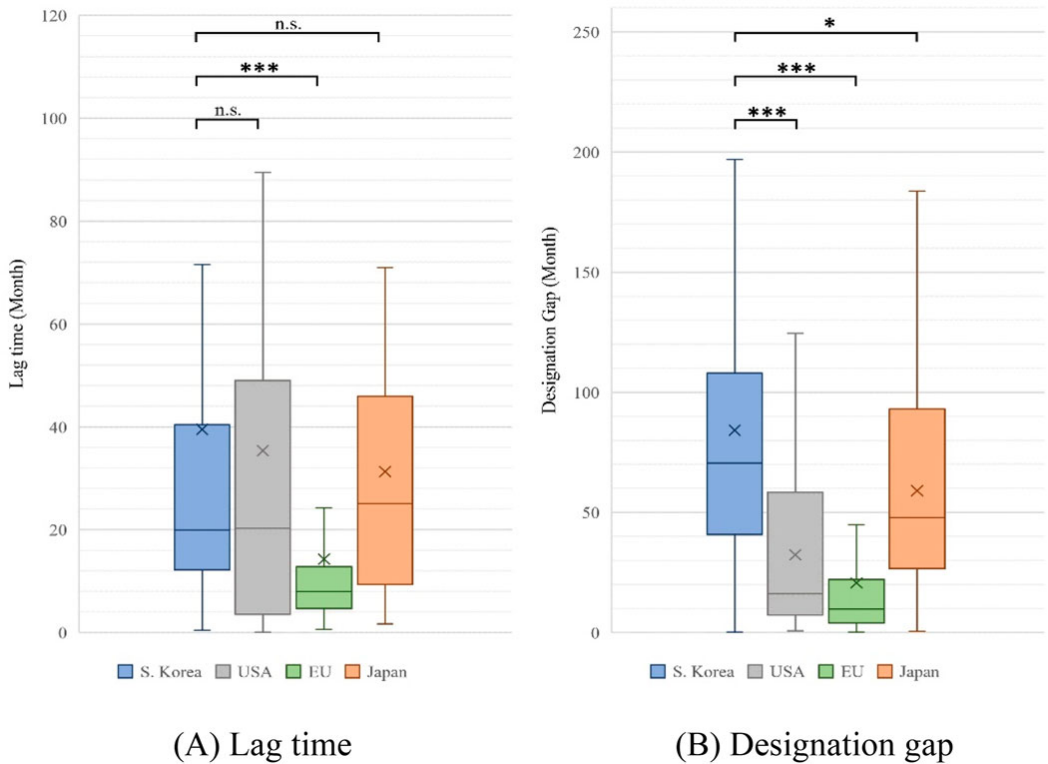
Lag time and designation gap of orphan drugs in each jurisdiction, excluding the first approved and designated product. Note: Based on 169 products designated as orphan drugs in South Korea from 2012 to 2021. We compared the lag time and designation gap by country for products approved in each country, excluding the first-approved and designated products. (A) Lag time: South Korea vs. USA (*P* = 0.503), the EU (*P* < 0.001), and Japan (*P* = 0.903). (B) Designation gap: South Korea vs. USA, EU (*P* < 0.001), and South Korea vs. Japan (*P* = 0.011). (We marked n.s.: not significant, *P < 0.05, **P < 0.01, and ***P < 0.001).

Moreover, in the analysis of lag time by each jurisdiction, excluding the first approved drugs in each jurisdiction, the results were as follows: South Korea (mean: 39.51 months, median: 19.96 months), USA (mean: 35.37 months, median: 20.23 months), EU (mean: 14.29 months, median: 7.97 months), and Japan (mean: 31.30 months, median: 25.13 months). There were no statistically significant differences among South Korea, the USA, and Japan (USA: *P* = 0.503, Japan: *P* = 0.903), but EU had a significantly lower lag time than South Korea (*P* < 0.001) (Table 4) (Figure 2).

**Table 4.** Lag time and designation gap of orphan drugs in each jurisdiction, excluding the first approved and designated product.

	Jurisdiction	N	Mean	SD	Median	Min – Max	*P* value
Lag time	South Korea	95	39.51	57.62	19.96	0.46–324.04	–
	USA	33	35.37	40.69	20.23	0.10–140.46	0.503
	EU	60	14.29	24.17	7.97	0.66–153.90	<0.001
	Japan	58	31.30	29.57	25.13	1.71–152.26	0.903
Designation gap	South Korea	101	84.23	64.36	70.53	0.26–369.45	–
	USA	28	32.35	34.37	16.18	0.66–124.64	<0.001
	EU	62	20.68	29.59	9.75	0.20–150.19	<0.001
	Japan	63	59.09	42.78	47.97	0.52–183.82	0.011

Note: Based on 169 products designated as orphan drugs in South Korea from 2012 to 2021. First, products approved in lag time and first designated products in the designation gap are excluded. Lag time: Period (months) from the first country of approval to the approval of drugs by each country for products approved in each country. Designation Gap: Period (months) from the first country of designation to the designation of drugs by country for products approved in each country. N: Number of designated (or approved) products in each country; SD: Standard Deviation. *P* value: Compared with South Korea.

In the analysis of designation gap by each jurisdiction, excluding the first approved drugs in each jurisdiction, the results were as follows: South Korea (mean: 84.23 months, median: 70.53 months), USA (mean: 32.35 months, median: 16.18 months), EU (mean: 20.68 months, median: 9.75 months), and Japan (mean: 59.09 months, median: 47.97 months). The designation gap in South Korea significantly differed from that of other countries (USA, EU: *P* < 0.001; Japan: *P* = 0.011) (Table 4) (Figure 2).

### Analysis of approval time, lag time, and designation gap of orphan drugs approved in all jurisdictions

In all the four countries, 45 items were designated and approved as orphan drugs, and approval and lag times, and designation gap were analysed by jurisdiction.

**Figure 3.**
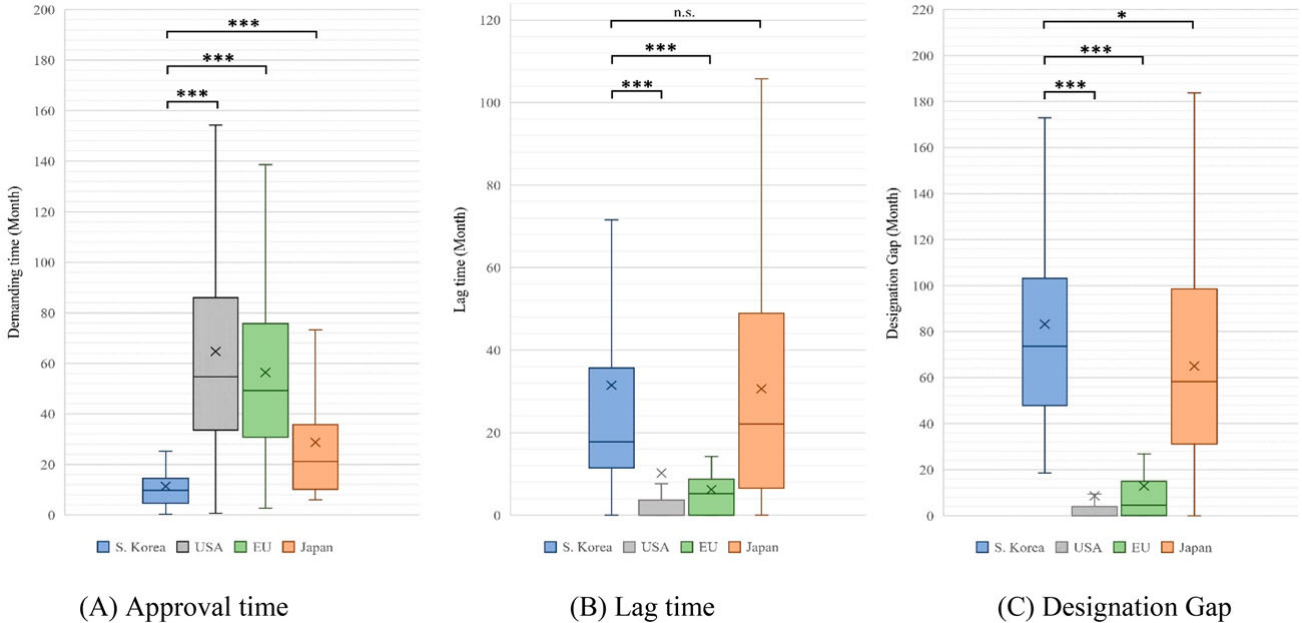
Approval time, lag time, and designation gap of orphan drugs approved in all jurisdictions. Note: Based on 169 products designated as orphan drugs in South Korea from 2012 to 2021. We compared the demand time, lag time, and designation gap by country for 45 products approved in all countries. (A) Approval time: South Korea vs. USA, EU, and Japan (*P* < 0.001). (B) Lag time: South Korea vs. USA and EU (*P* < 0.001), and South Korea vs. Japan (*P* = 0.812). (C) Designation gap: South Korea vs. USA, EU (*P* < 0.001), and South Korea vs. Japan (*P* = 0.048). (We marked n.s.: not significant, *P < 0.05, **P < 0.01, and ***P < 0.001).

The results of the approval time analysis were as follows: South Korea (mean: 11.38 months, median: 9.70 months), USA (mean: 64.75 months, median: 54.70 months), EU (mean: 56.37 months, median: 49.20 months), and Japan (mean: 28.71 months, median: 21.20 months). The approval time for South Korean drugs was significantly different from that for other countries (USA, EU, Japan: *P* < 0.001) (Table 5 and Figure 3). The results of lag time analysis were as follows: South Korea (mean: 31.48 months, median: 17.82 months), USA (mean: 10.22 months, median: 0.00 months), EU (mean: 6.18 month, median: 5.19 months), and Japan (mean: 30.61 months, median: 22.04 months). While there was no statistically significant difference between South Korea and Japan (*P* = 0.812), South Korea showed significant differences compared with the USA and EU (USA, EU: *P* < 0.001) (Table 5 and Figure 3). The results of designation gap analysis were as follows: South Korea (mean: 83.21 months, median: 73.65 months), USA (mean: 8.59 months, median: 0.00 month), EU (mean: 12.92 months, median: 4.49 months), and Japan (mean: 65.01 months, median: 58.37 months). South Korea showed a significant difference compared with the other countries (USA, EU: *p* < 0.001; Japan: *P* = 0.048) (Table 5 and Figure 3).

**Table 5.** Approval time, lag time, and designation gap of orphan drugs approved in all jurisdictions.

Country	Approved products (N)	Approval time			Lag time			Designation gap		
		Mean	Median	*P* value	Mean	Median	*P* value	Mean	Median	*P* value
South Korea	45	11.38	9.70	–	31.48	17.82	–	83.21	73.65	–
USA		64.75	54.70	<0.001	10.22	0.00	<0.001	8.59	0.00	<0.001
EU		56.37	49.20	<0.001	6.18	5.19	<0.001	12.92	4.49	<0.001
Japan		28.71	21.20	<0.001	30.61	22.04	0.812	65.01	58.37	0.048

Note: Based on 169 products designated as orphan drugs in South Korea from 2012 to 2021. Approval time (months): Period from designation to approval of drugs by country for the 45 products approved in all countries. Lag time (months): Period from the first country of approval to approval of drugs by country for the 45 products approved in all countries. Designation gap (months): Period from the first country of designation to the designation of drugs by country for the 45 products approved in all countries. N: Number of approved products in all countries *P* value: Compared with South Korea.

## Discussion

In this study, the availability indicators of drugs designated and approved as orphan drugs in South Korea were analysed by comparing them with those in the USA, EU, and Japan. Over the last 10 years (2012–2021), the approval rate of drugs designated as orphan drugs was 11.22% (395/3,521) in the USA and 6.31% (82/1,300) in EU, which was very low compared with that in South Korea (68.64% [116/169]) and Japan (67.94% [214/315]). Although the USA approval rate was lower than those of South Korea and Japan, the number of approved drugs was the highest in the USA, and in EU, both the approval rate and number of approved items were the lowest.

These results can be attributed to the tendency of the USA and EU to designate many drugs as orphans in the early stages of R&D. That is, many drugs are designated as orphan drugs, and a considerable amount of time is required to approve the development process of preclinical and clinical trials. However, in South Korea and Japan, drugs that have already completed R&D abroad or in their own jurisdiction are designated as orphan drugs; therefore, the number of orphan drug designations is low and the approval rate is high. The results of the analysis of the approval rate for 169 drugs designated as orphan drugs in South Korea showed that the USA had the highest approval rate at 92.31% (120/130), while South Korea had the lowest at 68.64% (116/169). In addition, the USA had the highest number of first approved products (87), showing the best availability to orphan drugs.

The results of the approval time analysis showed that the USA and EU required more time from the designation of orphan drugs to approval than South Korea and Japan, which could also be attributable to the tendency of the USA and EU to designate drugs as orphan in the early stages of development.

Drug lag time analysis showed that the lag times in South Korea and Japan were longer than those in the USA and EU, which is consistent with the findings of previous studies. Due to the comparatively active R&D of orphan drugs in the USA and EU, many drugs have been approved for the first time in each country, while in South Korea and Japan, even for orphan drugs that have already been approved abroad, drug lag time could be prolonged depending on the global pharmaceutical company’s marketing approval plan. In addition, as found earlier, the USA had the largest number of first approved orphan drugs and a short drug lag time, despite having the longest approval time from designation among the four countries, suggesting that the active R&D of orphan drugs had a positive effect on patient availability. Excluding the first approved drugs from each country, the lag time and designation gap were shorter in the EU than those in other countries, which could be interpreted as orphan drugs developed abroad being rapidly designated and approved as orphan drugs. That is drug availability to patients was higher in EU than in South Korea and Japan, as the designation and approval application for orphan drugs developed overseas was quickly established.

Our findings showed that both the designation gap and lag time in South Korea and Japan were longer than those in the USA and EU, which implies that in South Korea and Japan, the drugs are designated and approved after a considerable amount of time, even when they had already been designated and approved as orphan drugs in other countries, suggesting a correlation between designation gap and lag time.

The difference in availability indicators for orphan drugs by jurisdiction is the result of a combination of various factors, such as the new drug development and healthcare environments, as well as differences in policies related to orphan drugs. In the USA and EU, the designation of orphan drugs can be actively carried out even in the early stages because of the abundant resources available for new drug development. Active policy support has created an environment that leads to the success of the development of orphan drugs. Therefore, even orphan drugs in the early stages of development with insufficient clinical trial data can be designated as orphan drugs if certain conditions are met; thus, R&D funds as well as administrative support can be received. Therefore, it is possible to apply for products to become orphan drugs with preclinical trial data, even for pharmaceuticals in the early stages of development without clinical data. The Office of Orphan Product Development (OOPD) in the USA and the European Committee for Orphan Medicinal Products (COMP) in EU provide integrated administrative support through direct communication with pharmaceutical companies (EMA, 2023b; FDA, 2022b; GAO, U. S., 2018).

However, in Japan, the criteria for the prevalence of diseases designated as orphan drugs are stricter (less than 50,000 people), and clinical trial or development feasibility data showing that the effect of the drug is medically superior to that of alternative drugs are required. Even in procedural aspects, since drugs have to go through multiple analyses and approvals, including at MHLW, PMDA, and the Committee for New Drugs, it is difficult to designate the drugs as an orphan drug in the early stages where the developmental aspect is unclear (Loorand-Stiver et al., 2013; Maeda et al., 2017; Nagaraja et al., 2020).

In South Korea, the data required for the designation of orphan drugs are relatively simple, and the procedure is simplified. However, compared to other countries, an application for the designation of orphan drugs is made only when the application for approval for commercialisation is imminent, which is attributed to the benefits in the regulatory process provided by the designation of an orphan drug. In particular, in South Korea, in accordance with the Rare Disease Control Act, which was enacted in 2016, benefits such as tax incentives for the development of orphan drugs, priority review, exemption from submission documents in approval review, and exclusivity after approval are granted. However, the benefits are concentrated in the process after the commercial approval process or after approval. That is, the benefits are concentrated in the later stages, when development has progressed considerably, rather than promoting the R&D of orphan drugs. Therefore, as the environment continues to rely on imported orphan drugs, the drug lag may intensify, which is a similar phenomenon occurring in Japan, which has an orphan designation and approval process similar to those of South Korea (Cho & Han, 2022; Miyazaki et al., 2022).

Although South Korea has established an institutional means to support orphan drugs in the early stages of development by designating orphan drugs in the development stages, there have been no cases of commercialisation through such support. There were many cases in which products from Korean pharmaceutical companies are designated as orphan drugs first in the USA or EU rather than in South Korea, and only two of them were designated as development-stage orphan drugs by the Korean Ministry of Food and Drug Safety (KMFDS) as of July 2022, which is a manifestation of the current status of South Korea (Sujin Gwak, 2019).

In the USA, to provide simple benefits, such as tax support for orphan drug development, the FDA established and led the Rare Disease Cure Accelerator, Data Analytics Platform (RDCA-DAP) with patient groups, accumulating genetic information of patients with rare diseases. Genetic information of patients with rare diseases can be used in the development of orphan drugs (Barrett et al., 2023). In addition, to solve the difficulties in recruiting participants for clinical trials, active policy support is being expanded, including revision of related guidelines to use real-world evaluation (RWE) and real-world data (RWD) for the approval of orphan drugs (FDA, 2018). As multifaceted efforts are being made globally to promote the development of orphan drugs, active attention to the related trends is required.

In this study, quantitative indicators from the designation to approval stage for orphan drug availability, i.e. approval time, lag time, and designation gap, were analysed and compared internationally. However, this study had a limitation in that the dataset was limited to products designated and approved as orphan drugs in South Korea and did not include all orphan drugs approved in each jurisdiction. In addition, since most orphan drugs are expensive, reimbursement from public insurance can be considered a very important factor in accessibility; however, issues related to reimbursement were not included because they were outside the scope of this study.

## Conclusion

Our findings showed that orphan drug availability indicators in South Korea are not as favourable as those in major developed countries, which can be fundamentally attributed to the lack of active development of orphan drugs in the early stages. Therefore, to fundamentally improve access to orphan drugs in South Korea, a regulatory science policy that can comprehensively support both early-stage R&D and commercialisation is required.

## Author contributions

EY Shin, YJ Hong, KM Lim, and TH Kim contributed to the study design, data collection, statistical analysis, manuscript development, and review, under the guidance of JH Lee. JH Lee supervised the entire study, from study design, data collection and analysis, and manuscript editing to manuscript submission. All the authors have read and approved the final version of this manuscript.

## Data Availability

The datasets analysed in the current study are available from the corresponding author upon request.
